# Navigating through digital folders uses the same brain structures as real world navigation

**DOI:** 10.1038/srep14719

**Published:** 2015-10-01

**Authors:** Yael Benn, Ofer Bergman, Liv Glazer, Paris Arent, Iain D. Wilkinson, Rosemary Varley, Steve Whittaker

**Affiliations:** 1 University of Sheffield, Dept. of Psychology, Western Bank, Sheffield, S10 2TP, UK.; 2 Bar-Ilan University, Dept. of Information Science, Ramat Gan, 5290002 Israel; 3 University of Sheffield, Academic Unit of Radiology, Royal Hallamshire Hospital Glossop Road, Sheffield, S10 2JF; 4 University College London, Division of Psychology and Language Sciences, 2 Wakefield Street, London, WC1N 1PF, UK.; 5 University of California at Santa Cruz, Dept. of Psychology, CA 95064

## Abstract

Efficient storage and retrieval of digital data is the focus of much commercial and academic attention. With personal computers, there are two main ways to retrieve files: hierarchical navigation and query-based search. In navigation, users move down their virtual folder hierarchy until they reach the folder in which the target item is stored. When searching, users first generate a query specifying some property of the target file (e.g., a word it contains), and then select the relevant file when the search engine returns a set of results. Despite advances in search technology, users prefer retrieving files using virtual folder navigation, rather than the more flexible query-based search. Using fMRI we provide an explanation for this phenomenon by demonstrating that folder navigation results in activation of the posterior limbic (including the retrosplenial cortex) and parahippocampal regions similar to that previously observed during real-world navigation in both animals and humans. In contrast, search activates the left inferior frontal gyrus, commonly observed in linguistic processing. We suggest that the preference for navigation may be due to the triggering of automatic object finding routines and lower dependence on linguistic processing. We conclude with suggestions for future computer systems design.

Personal Information Management (PIM) is an activity in which individuals organize personal (often digital) information for later retrieval. The two main ways by which digital information items such as personal files can be retrieved are *navigation* and *search*. When ‘navigating’, users manually traverse their organizational hierarchy until they reach the folder in which the target item is stored. For search, users first generate a query specifying an attribute of the target item, and when the search engine returns a set of results, they select the relevant item^1^.

Navigation is dependent on virtual folder structure. A ‘virtual folder’ is a visual metaphor for a location, borrowed from physical information management systems. As such, personal computer users can see and manipulate items “inside” folders; e.g., drag and drop contents from one folder to another. During decades of folder use, this organizational method has received much criticism: folders conceal information items, reducing the chances of opportunistic encounters with critical files^2^, and categorization is cognitively difficult because items may relate to multiple folders^3^. Most importantly, folders and the location metaphor force users to remember the exact path to a specific information item, which can be difficult, especially if time has elapsed between storing and retrieving the item^4^. Compared with navigation, search is more flexible, allowing users to retrieve an item using any attribute they happen to remember, such as a word it contains, modification date, partial file name, etc.^4^.

These benefits, along with significant recent improvements in desktop search engines^4^,^5^,^6^,^7^ should induce strong user preferences for search over navigation. However, research consistently shows that users prefer navigation over search e.g.^8^,^9^,^10^, and that search is often used only as a last resort^1^.

Why do people prefer navigation, despite its apparent limitations compared with search? In previous work^1^ we proposed several possible explanations, including that users are familiar with their own folder structure which stays relatively stable over time. By contrast, the flexibility of search may compromise consistency as users are able to retrieve the same file using a different search terms.

Here we explore the neurocognitive basis of navigation and search in personal information management, hypothesizing that people prefer virtual folder navigation because of its similarities to navigation in the real-world. We predict that the same brain structures involved in navigating physical environments will be employed in virtual navigation. These structures have been identified in animals^11^,^12^,^13^ and humans^14^,^15^,^16^, and include regions of the posterior limbic system, such as the parahippocampal gyrus^17^,^18^ and the retrosplenial cortex^19^. By contrast, search competes for linguistic resources in working memory. Support for this view comes from Bergman *et al.*^20^, who found that searching interfered more than navigation with a concurrent task of maintaining words in working memory. Generating a search term is likely to engage cortical structures within the left fronto-temporal region, which are typically associated with linguistic and executive processing. We test these hypotheses using functional magnetic resonance imaging (fMRI).

We collected whole-brain functional images from 17 healthy right-handed participants while they engaged in file retrieval using the standard Windows 7 Operating System. To increase ecological validity, participants retrieved their own files from their personal laptop. Prior to the study, a list of recently used file names was extracted from their laptop. During the scanning session, these file names were used as target items and participants were instructed to retrieve the file from their laptops using search or navigation as instructed. Two control tasks, one for search and one for navigation, were included. The control tasks used visual and motor activities matched to the search and navigation tasks, but did not engage core cognitive processes involved in the experimental conditions (e.g., memory for virtual location or file attributes).

During navigation, participants traversed their own file structure to reach the given target file (Fig. 1a). The matched control task (henceforth *control navigation*) involved a phantom file system with phantom folder and file names. To subtract neural processing associated with folder traversal and selection, participants were given a letter (e.g., ‘f’) and asked to traverse the phantom file system by clicking on folders starting with that letter (e.g., ‘flower’, ‘feather’, etc.) (Fig. 1c). The task was successfully completed when participants reached a file called ‘press done’.

In the search condition, participants were required to locate the target file by typing a search query into the search bar of the standard Explorer window using a mouse and an on-screen keyboard (Fig. 1b). Given that the target file name was presented, participants were instructed to use any query term other than the file name, as simply copying the target name would have bypassed the cognitive systems necessary for generating meaningful search terms. The matched search control task (henceforth *control search*) required participants to generate an association to a stimulus word. These stimuli were selected from a list of words with strong associative partners (e.g., in response to the word ‘black’, most people respond with ‘white’ (50%), followed by ‘red’ (14%) and ‘dark’ (5%))^21^. These highly predictable associations were chosen to minimize effort in generating a word. After participants typed an associative response using the online keyboard, a new Explorer window with a list of phantom file names appeared that included the word that the participant had just typed (Fig. 1d). To mirror selection of targets from a search results list, participants were required to locate the file labeled with the word they had just entered (e.g., if they entered ‘box’ in response to ‘black’, they had to select ‘box.txt’) and click on it with the mouse.

Behavioral data were extracted from screen recordings of each laptop collected during the experiment. Data included success rate on the different tasks, and the average depth of structure used by participants to store files. The mean folder-depth structure was 2.54 (SD = 0.82), similar to previous findings^22^. The mean number of trials per block were as follows: search: *M* = 2.02, *SD* = 0.65; navigation: *M* = 3.50, *SD* = 1.86 control search: *M* = 4.16, *SD* = 1.15; control navigation: *M* = 4.31, *SD* = 2.39. The mean success rate on the different tasks (i.e., the percentage of the trials resulting in identification of the target correctly within the 30 seconds time block) was as follows: search: *M* = 28.48%, *SD* = 18.58%; navigation *M* = 27.25% *SD* = 13.05%; control search: *M* = 64.47%, *SD* = 13.28% and control navigation: *M* = 54.18%, *SD* = 14.38%. There were no significant differences in behavioral results between male and female participants, and for subsequent analyses data were collapsed into a single group.

Neuroimaging group analysis of the contrast between the search condition and its control, using a *p* value of 0.001, a minimum cluster size of K = 10, and False Detection Rate (FDR) correction at cluster level of *p* < 0.05 revealed activations in and around Broca’s area (BA 44/45), left middle frontal gyrus, left precuneus and superior parietal lobule (BA 7, 19) (Table 1).

The contrast between the navigation condition and its control using, *p* < 0.001, k = 10, and FDR correction at cluster level of *p* < 0.05, revealed large bilateral activations in posterior regions, including the retrosplenial cortex, parahippocampal gyrus, cingulate and lingual gyri, cuneus, precuneus and left superior parietal lobule. Activation was also observed in the left middle and superior frontal gyri (BA 6/8). Activation of the parahippocampal gyrus was more posterior to the region previously identified as the Parahippocampal Place Area (PPA)^14^, but lay within a region that has been associated with spatial navigation^17^.

The contrast between the navigation and search conditions (navigation—control navigation) > (search—control search) using *p* < 0.001, k = 10, and FDR correction at cluster level of *p* < 0.05, revealed activations only in posterior regions of the brain, and included the cuneus, precuneus and posterior cingulate gyrus (Table 1). The contrast between the search and navigation conditions (search—control search) > (navigation—control navigation) revealed no significant areas of activation.

In sum, while search resulted in strictly left lateralized activation in areas strongly associated with linguistic and working memory processing, navigation resulted predominantly in bilateral activation of posterior regions of the brain, associated largely with real-world navigation, retrieving information from memory, and low-level sensory-perceptual processing (Fig. 2).

The contrasts directly comparing the search and navigation tasks further reinforce the observation that navigation relies largely on posterior structures and right-hemisphere mechanisms. However, we note that no significant differences are revealed by the (search—control search) > (navigation—control navigation) contrast. This may be due to some overlap between the two tasks. For example, when people search for a file, they may also have an idea of its location, and when a user navigates to a file, this is likely to require some linguistic engagement such as comparing file names within the target folder. This could be explored in future studies.

Our results, combined with evidence from^20^, provide a possible explanation of user preference for navigation over search when retrieving items from digital personal information systems. Virtual navigation for digital files involved bilateral activation of posterior brain regions which are also engaged during navigation in the physical world^14^,^15^,^16^,^17^,^18^,^19^, and in which the level of theta activation has been shown to predict performance in navigating to a specific target^23^. These same brain areas are also used for navigation by monkeys^11^, rats (for a review:^12^,^19^) and pigeons^13^. Combined with the observation that patients with severe language difficulties following brain damage perform well on navigational tasks^24^, we conclude that both virtual and real-world navigation can be performed with minimal dependence on linguistic processing. Search, on the other hand, involved left lateralized, mostly frontal activations that are associated with linguistic (Broca’s area) processes. These activations were not due to basic lexical retrieval as our control task subtracted activations related to such processing. Instead left inferior frontal and left parietal activations were likely to be linked to the attentional and strategic planning demands of linguistically controlled processing, necessary for isolating precise search terms^25^,^26^.

The need to actively generate a search strategy may divert linguistic and attentional resources to this task, which may explain why users choose to use search only as a last resort despite its flexibility^1^. Activation of the left middle/superior frontal gyrus was observed during navigation, suggesting that navigation also requires a degree of executive and working memory resources^27^. However, these activations did not extend to linguistic structures, strengthening the argument that navigation requires minimal language mediation.

A few limitations of the current work should be addressed in future studies. First, having nine female and eight male participants meant that we could not reliably explore potential gender differences in brain activations associated with search and navigation. Gender differences have previously been reported with relation to real world navigation^28^, and future research should explore whether these can also be observed in virtual folder navigation. Second, humans differ in their spatial navigation abilities, and engage different brain structures to perform such tasks, depending on the strategy they employ^29^. Future research should investigate, by combining behavioral measures, whether navigational strategies in the real world affect activations related to virtual folder navigation. Previous fMRI studies of navigation or place identification have tended to employ a localizer task to identify the critical neural circuits in individual participants^14^,^30^,^31^. Future studies could include a localizer task that involves real-world navigation (e.g., maze navigation), to explore the extent to which common areas are engaged in virtual folder and real navigation, and whether these differ according to individual strategies. Finally, our study adopted a block design with a 3 second fMRI resolution, which meant that it was not possible to explore brain activations linked to the various sub-components of each task, e.g., generating a search term vs. scanning the file list returned by the search query. Future studies may wish to employ other methodologies such as EEG to enhance the temporal resolution, allowing inferences about these subtasks.

The current findings offer a neural explanation for the repeatedly documented preference for navigation over search. They further explain why this preference has not altered despite advances in search engine technology and performance, and predict that this preference is unlikely to change with further improvements in search technologies. Instead of attempting to replace navigation with search^5^,^32^, we suggest a user-subjective approach to PIM systems design^33^. This design approach takes folders as given, and attempts to exploit and improve them rather than replace them. We argue that humans have developed mechanisms that allow them to retrieve an item from a specific location (be it real or virtual), by navigating the same path they followed in storing the information. These deep-rooted neurocognitive biases lead to automatic activation of retrieval routines, which have minimal reliance on linguistic processing, leaving the language system available for other tasks.

## Methods

Ethical approval for the study was granted by the University of Sheffield, Department of Psychology ethics committee. The research was conducted in a manner consistent with the American Psychological Association’s ethical principles, and was carried out in accordance with the approved guidelines. All participants were informed of the goal and nature of the study and signed a consent form prior to taking part.

### Participants

Twenty healthy right-handed students (11 females), from various disciplines (all were in their second year of undergraduate degree or above, including post-graduate students), aged between 19–29 years (mean = 22.8, SD = 2.87), took part in exchange for a small financial reward. Three participants were excluded from analysis due to either technical problems or for not complying with the instructions, leaving data from 17 participants (9 females).

### Apparatus and materials

One day prior to the imaging experiment, the experimenter installed Python programming language with Pygame 1.9.1 add-on (http://www.pygame.org), and Debut screen-capture software (http://debut-video-capture.en.softonic.com) onto the participant’s laptop to enable running the paradigm, and recording of screen activity during the experiment. To ensure that participants used the same up-to-date search engine, their laptops had to run Windows 7. During this session, the experimenter also extracted a list of recently used files (*.doc, *.xls, *.pdf, *.ppt) from the participant’s laptop. This list was used by the experiment program to display target file names during the navigation and search tasks (file names were randomly assigned to these tasks). Only recently accessed files were used, to overcome potential confounds of file age and individual variability in engagement with their system. During this preparatory meeting, participants practiced the experimental tasks (including the two control conditions).

The fMRI session took place the next day. The participant’s laptop was connected to a high-resolution LCD screen in the scanner room. The screen was visible to the participant in the scanner via a mirror positioned at the top of the radiofrequency head coil. Participants interacted with their laptop using an MRI-compatible optical mouse (NAtA technologies, FOM-2B-10B fMRI Mouse) connected to the laptop via fMRI-compatible USB cable. For typing, participants used an on-screen keyboard available from Windows 7 accessories.

### Stimuli

**Figure 1:**
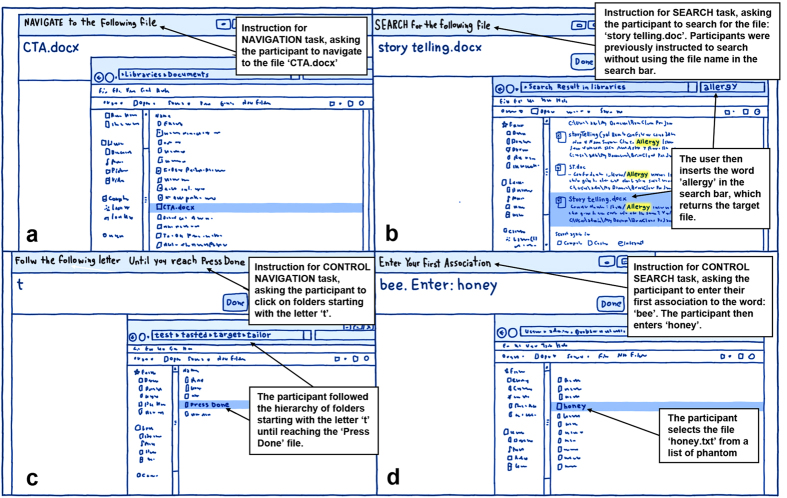
An illustration of the Instruction and Explorer windows in all conditions. (**a**) navigation task, (**b**) search task, (**c**) control navigation task, (**d**) control search task.

**Figure 3:**
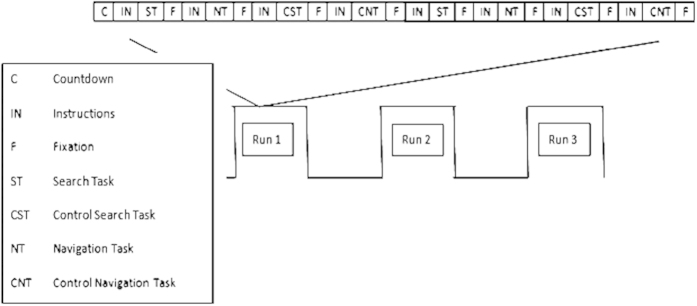
Illustration of experiment design.

The paradigm involved a block-design with four conditions of 30 seconds each. There were two experimental conditions: navigation and search, and two control conditions matched to each experimental condition. In all conditions, two windows opened on the screen: an instruction window and the standard Windows Explorer window (Fig. 1). All conditions began with an instruction slide for 6 seconds informing the participant about the task ahead (e.g. ‘SEARCH for the following file’ or to indicate the search control task: ‘Enter your first association to the following word, then select the file with the same name’), and ended with a 3 second fixation slide (Fig. 3). We used a within subjects design so that each participant accessed files from their same personal file system but using different strategies (i.e., navigation and search) in a controlled and randomized manner.

In both experimental conditions, the target file name appeared in the instruction window. Participants were then required to either locate the target using hierarchical navigation within the Explorer window, or by entering a search term in the search bar of the Explorer window. Given that the file name was presented to participants, they were instructed to refrain from using the file name or parts of it in their search query. Instead, participants were given examples of other information they could use, such as words in the file or search by the date they last accessed or created it. In practice, none of the participants used the creation or access date, and instead all used words that appeared in the file but not in the file name. For example, when searching for a file ‘Memory reading’ Participant 3 typed ‘Baddeley’ in the query box because she recalled that the file included Baddeley’s paper on working memory; and when Participant 5 searched for a file called ‘Simone Weil fact file’ she searched for the word ‘Paris’ because she knew it contained the information that Simone Weil was born in Paris. Once participants reached the target file either by search or navigation, they were required to click on it once and then press the ‘done’ button in the instruction window. The experiment block ended after 30 seconds regardless of whether the target file was located. If the target file was located within less than the 30 seconds of the length of a block, the instruction window displayed a new target, and the Explorer window was re-set.

The control tasks had similar interfaces to the experimental tasks, to allow for the subtraction of visual, motor and superficial levels of cognitive processing such as basic decision-making (e.g., deciding which folder to click on), or word-generation (i.e., generating a target word). In the control condition for the navigation task, the instruction window presented a target letter, and the Explorer window contained a list of randomly generated folders (Fig. 1c). Participants were required to traverse the hierarchy of folders, by clicking on folders that began with the target letter. Each trial came to an end once participants reached a file called ‘press done.txt’. They were required to highlight the file, and then press the ‘done’ button in the instruction window. If the 30 seconds allotted to the task were not over, a new target letter would be presented, with a new set of randomly generated folders in the Explorer window. The traversing tree depth was randomly generated between a depth of 2 and 7 levels. This is based on^22^, which indicated that the average tree-depth is 2.86, with SD of 1.85.

In the control search task, the instruction window presented a randomly selected word from a list of words with strong associates^21^. Participants were required to type their first association to this word into the instruction window, and press the ‘ok’ button. A new Explorer window with a list of file names then appeared, including the word the participant just entered (Fig. 1d). Participants were required to locate that word, click on it, and press the ‘done’ button on the instruction window.

### Experiment Design

The experiment used a block design, with three runs (Fig. 3). Each run started with a 21 second countdown, followed by a six second fixation period, where participants were instructed to focus on a red cross located in the center of a black screen. All four conditions appeared twice in each run in semi-randomized order (Fig. 3). Each run lasted approximately 7 minutes, but this varied across laptops, due to variations in computer processor speed (*M* = 6 min and 38 seconds, *SD* = 39 seconds, longest: 7 minutes and 57 seconds, shortest: 6 minutes and 6 seconds). All search, navigation and control conditions terminated after 30 seconds, even if the target was not located. However, if the target was reached in less than 30 seconds, a new target was presented.

### Data Acquisition

All MR images were acquired at 3T (Ingenia 3.0T, Philips Healthcare, Best, Holland) using a fifteen-channel radiofrequency receive-only head coil. Cerebral vascular response to the tasks was recorded using the BOLD (blood oxygenation level-dependent), T2*-weighted signal time-course. During each functional scan, a time series of 153 dynamic datasets were obtained using a 2-dimensional single-shot, echo-planar imaging (EPI) sequence. The EPI scan parameters were as follows: repetition time (TR) =  3000 ms; echo time (TE) = 35 ms; sensitivity-encoding factor = 1.8; flip angle = 90°; in-plane voxel size = 2.4 mm × 2.4 mm interpolated to 1.8 mm × 1.8 mm; 35 contiguous 2-dimensional transaxial slices each having slice thickness = 4mm.

### Data Processing

All data analysis was performed using SPM8 (Wellcome Department of Imaging Neuroscience, London; www.fil.ion.ucl.ac.uk/spm/) implemented in Matlab (The MathWorks Inc., Natick, MA). Functional images were corrected for head movement. Realigned images were then spatially normalized to the standard SPM EPI template. Following normalization, images were smoothed using an 8 mm full-width half-maximum (FWHM) Gaussian filter.

To construct the individual design matrix, screen recordings obtained from each laptop during the fMRI study were used to extract exact timings and behavioral compliance with the task. This was done by viewing the screen recordings, and marking the exact number of seconds from the beginning of the scan (i.e., when the experiment started on the laptop) to the beginning and end of each of the experimental and control tasks, as well as the fixation and instruction periods. Behavioral data were extracted in a similar manner, by coding the depth of folder structure, successful vs. unsuccessful search and navigation tasks (i.e., whether the target was reached within the time limit), and the total length of scan time. Coding of information was done independently by two coders (the third and fourth authors), and any inconsistencies were checked and resolved. After specifying the design matrix for each participant (using timing and behavioral data from the recorded screens), the BOLD signals induced by different conditions were assessed using a general linear model. Contrasts were constructed for each individual, and then used for the second level analysis.

## Additional Information

**How to cite this article**: Benn, Y. *et al.* Navigating through digital folders uses the same brain structures as real world navigation. *Sci. Rep.* **5**, 14719; doi: 10.1038/srep14719 (2015).

## Figures and Tables

**Figure 2:**
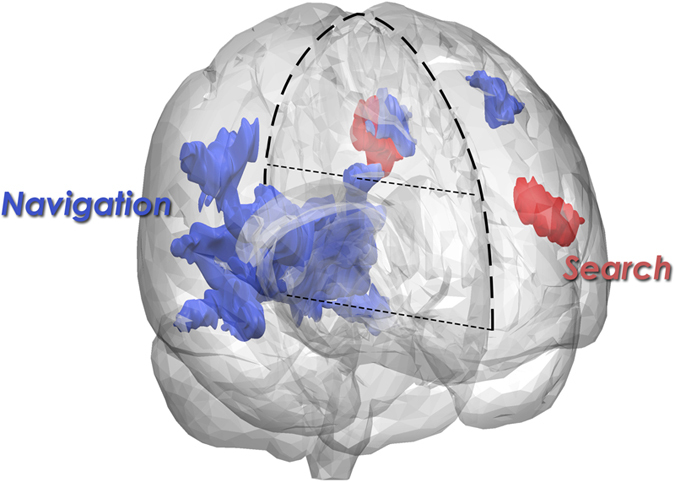
A 3D model illustrating bilateral posterior regions activated for folder navigation (blue), and left inferior frontal activation for search (red). Results uncorrected, p = 0.001, a minimum cluster size k = 10, and an FDR correction at cluster level of p < 0.05. The bold dotted line marks the external outline of the brain, while the two thin dotted lines mark the center-line, dividing between the left and right hemispheres. The figure was produced by creating a 3-D model of the standard MNI152 brain using the 3D Slicer open access software (www.slicer.org/). The 3-D activations were then constructed and placed in the model using the information provided by each of the 2-D slices on the X Y and Z planes as produced by SPM. Permission to publish under Open Access is granted by the authors who are copyright owners.

**Table 1:** Brain Regions Identified For Search and Navigation.

**Region**		**Broadmann Area**	**Max coordinate**			**Cluster-level FDR corrected *p***	**Z**	**k**
**Lobe**	**Anatomical localization of cluster**		**X**	**Y**	**Z**			
Search vs. control search (p = 0.001, k = 10, FDR cluster level correction *p* < 0.05)								
Parietal	Precuneus/superior parietal lobule (L)	7, 19	−30	−78	40	0.016	4.10	252
Frontal	Middle/Inferior frontal gyrus (L)	9, 44, 45	−48	14	18	0.016	4.91	243
Navigation vs. control navigation (p = 0.001, k = 10, FDR cluster level correction *p* < 0.05)								
Limbic/Occipital/parietal	Posterior cingulate (including the retrosplenial cortex (L,R), parahippocampal gyrus (L,R), cingulate gyrus (L,R) lingual gyrus (L,R), cuneus (L,R), precuneus (L,R)	18 (L,R), 23 (L,R), 29 (L,R), 30 (L,R)	20	−56	16	0.000	5.39	5563
Frontal	Middle/superior frontal gyrus (L)	6, 8	−24	14	62	0.039	4.86	164
Parietal	Precuneus (L), superior parietal lobule (L)	7, 19	−28	−64	48	0.039	4.23	181
(Navigation –Control Navigation) > (Search—Control Search) (p = 0.001, k = 10, FDR cluster level correction *p* < 0.05)								
Occipital/parietal	Cuneus, precuneus (R)	7 (R), 31 (R)	12	−68	30	0.051	5.01	163
Limbic/occipital/parietal	Posterior cingulate, cuneus, precuneus (L)	7, 81, 31	−10	−72	28	0.053	4.27	228
Limbic/Parietal	Cingulate gyrus (R), precuneus (L,R)	7 (L,R), 31 (R)	−4	−56	54	0.050	3.89	162

L = left; R = right; k = minimum cluster size.
